# Extensive Schmallenberg virus circulation in Germany, 2023

**DOI:** 10.1186/s13567-024-01389-5

**Published:** 2024-10-07

**Authors:** Kerstin Wernike, Luisa Fischer, Sönke Twietmeyer, Martin Beer

**Affiliations:** 1 Friedrich-Loeffler-Institut, Südufer 10, 17493 Greifswald - Insel Riems, Germany; 2 Wildlife Research Institute, State Agency for Nature, Environment and Consumer Protection North Rhine-Westphalia, Pützchens Chaussee 228, 53229 Bonn, Germany; 3 Eifel National Park, Urftseestraße 34, 53937 Schleiden-Gemünd, Germany

**Keywords:** Schmallenberg virus, bluetongue virus, arbovirus, biting midges, serology, wildlife

## Abstract

Schmallenberg virus (SBV) and bluetongue virus (BTV) are both transmitted by *Culicoides* biting midges and infect predominantly ruminants. To investigate the extent of virus spread in the 2022 and 2023 vector seasons, we serologically tested wild ruminants from western Germany. While antibodies against BTV were not detected in any animal, regardless of age or sampling time, numerous wild ruminants tested positive for antibodies to SBV. In 2022, a low seroprevalence of 4.92% was measured. In sharp contrast, 40.15% of the animals tested positive in 2023. Of the young animals, about 31.82% were seropositive, clearly indicating large-scale SBV circulation in summer and autumn 2023.

## Introduction, methods and results

The arboviruses Schmallenberg virus (SBV) and bluetongue virus (BTV) are significant pathogens affecting ruminants. Beside the similarities in host species, SBV and BTV have parallels in their transmission characteristics, as both are transmitted by *Culicoides* biting midges [1, 2]. SBV is an orthobunyavirus first identified in 2011 in cattle near the German-Dutch border [3]. In adult animals, an infection typically results in transient fever, diarrhea, and reduced milk production, while pregnant females may experience abortions, stillbirths, and severe congenital malformations in their offspring [1]. Since its emergence, SBV has spread rapidly across Europe. While the initial outbreaks from 2011 to 2013 were significant [4], subsequent years have seen a decline in reported cases, and finally a pattern of cyclical circulation with waves has been established, meaning that the virus re-appears to a greater extent every two to three years, while SBV is only sporadically detected in the intervening years [1, 5]. In contrast to the endemicity of SBV in the central European ruminant populations without any control measures, the orbivirus BTV has experienced significant fluctuations marked by outbreaks, periods of control and disease-freedom, and ongoing surveillance in the German-Dutch-Belgian region [6, 7]. The most recent outbreak occurred in autumn 2023, when BTV serotype 3 emerged in the Netherlands [8, 9] and spread within only a few weeks to Belgium, western Germany and even the UK. Bluetongue disease is characterized by fever, edema, hyperemia, hemorrhages, cyanosis and lameness, sometimes leading to the death of the animals [2]. Hence, both BTV and SBV induce major animal welfare issues and economic losses. Wildlife, particularly wild ruminants such as deer, might serve as reservoirs for both viruses, contributing to their persistence in a given region. In addition, wild ruminants are very suitable as sentinels to monitor the circulation of both viruses [6, 10].

Here, we investigated wild ruminant samples collected during the 2022/2023 and 2023/2024 hunting seasons in the Eifel National Park for the presence of antibodies against SBV and BTV. The Eifel National Park is a protected area which spans over 110 km^2^ located in the German federal state of North Rhine-Westphalia and bordering Belgium. In the 2022/2023 hunting season, blood samples were collected post-mortem from 61 animals (35 red deer, 26 roe deer), the sampling dates were between 24 October and 15 December 2022. Twenty-nine of the animals were juveniles (< 1 year), 22 were yearlings and 10 adults. In the 2023/2024 hunting season, blood samples were collected in the same region from 137 animals (92 red deer, 35 roe deer, 7 mouflon, 3 species unknown). Sampling dates were between 15 November and 14 December 2023. Sixty-six animals were juveniles, 36 yearlings, 31 adults and from four animals the age is unknown. All wildlife samples were analysed by the commercially available ID Screen Schmallenberg virus Competition Multi-species and ID Screen Bluetongue Competition ELISAs (both Innovative Diagnostics, Grabels, France) according to the manufacturer’s instructions.

**Figure 1:**
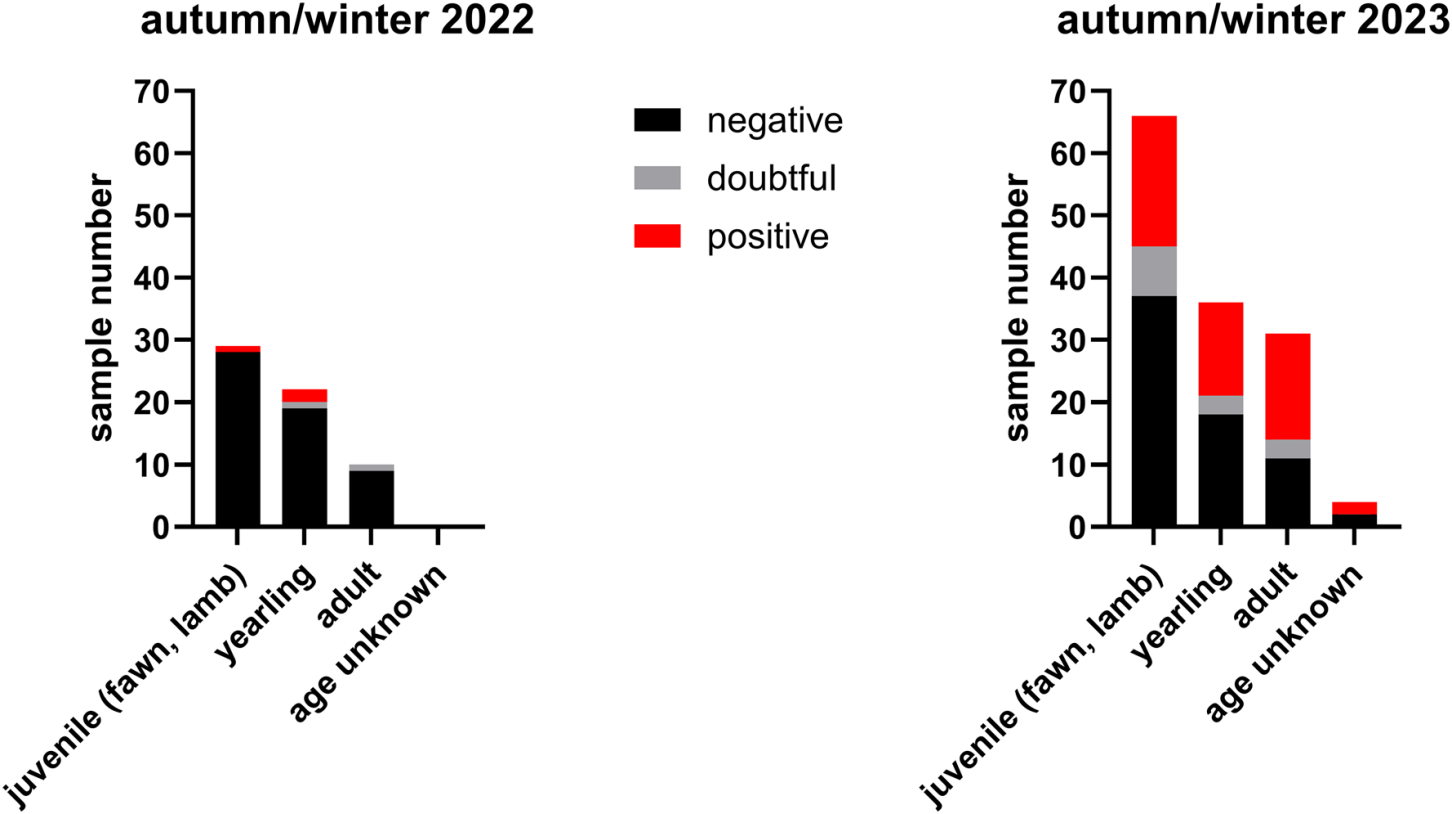
**Results of the wildlife sera separated by age categories for juveniles, yearlings and adults in the Schmallenberg virus antibody ELISA**. Positive results are shown in red, doubtful in grey and negative in black.

In the BTV antibody test, all samples gave negative results. In contrast to BTV, antibodies against SBV were detected in numerous wild ruminants. While in 2022 an overall seroprevalence of 4.92% (95% confidence interval (CI): 0–10.34%) was measured, 40.15% (95% CI: 31.94–48.35%) of the animals tested positive in 2023. Of particular note, in 2022 only one of the juveniles scored positive (1/29, 3.45%), but in 2023 about 31.82% (95% CI: 20.58–43.06%) of the juveniles displayed antibodies against SBV (Figure 1), which indicates a large-scale virus circulation in summer and autumn 2023 in the tested ruminant population.

## Discussion

The consistently negative results in the BTV antibody test, regardless of animal age or sampling time, suggest either none or very limited virus circulation, which is in line with the results of vector monitoring conducted in the same federal state in autumn 2023. Although several thousands of midges were tested, only a single BTV-positive midge pool was found [9]. Similarly, in the fall of 2023, after the virus was introduced into Germany from the Netherlands, only individual animals were positive on infected cattle and sheep farms in this region (source: German Animal Disease Reporting System TSN [11]). However, given the rapid and extensive virus spread in the Netherlands, it is expected that BTV continues to spread with the seasonal onset of vector activity in spring and summer 2024. Therefore, monitoring should be maintained in the mammalian host and/or insect vector populations.

In contrast to BTV, antibodies against SBV could be detected in numerous animals, though with variations between years. In wild ruminant samples collected in the German federal state North Rhine-Westphalia during the 2021/2022 hunting season, an overall seroprevalence of about 6.5% was reported [10], and one year later we found antibodies in only barely 5% of the animals. The impressively high increase in 2023 clearly demonstrates virus circulation to a large extent in the monitored region and confirms that SBV has established a pattern of cyclic re-circulation with waves. Consistent with the high number of infections of wild ruminants, SBV-induced malformation were seen in lambs in early lambing units at the beginning of 2024 in further European countries like the UK, and in this context a big outbreak was described [12]. In the preceding two years, i.e. 2021 and 2022, cases were reported only sporadically in the UK [12] and Germany (source: TSN [11]). However, the lack of systematic surveillance for SBV in livestock has resulted in a dearth of comprehensive serological data from farm animals that can be used to estimate the extent of virus circulation in these two and also further European countries. In the absence of systematic data from farmed ruminants, results of wildlife or vector monitoring are particularly valuable. Indeed, SBV was found in numerous biting midge pools collected in western Germany in autumn 2023 [9], further confirming our observation of large-scale SBV circulation. Unfortunately, data from midge testing in that region in 2021 or 2022 are not available for comparison, but seroprevalences in wild ruminants hint at very low levels of virus circulation. Therefore, we conclude that wild ruminants are suitable indicators for virus spread as has been shown also in recent years [13, 14], when farm animals or vectors responsible for virus transmission are not systematically monitored on a regular basis. The availability of continuous data on wildlife serology may assist in the estimation of the timing of the next wave of SBV circulation following years of declining seroprevalences.

## Data Availability

The datasets generated during the current study are available in the Zenodo repository [15].
